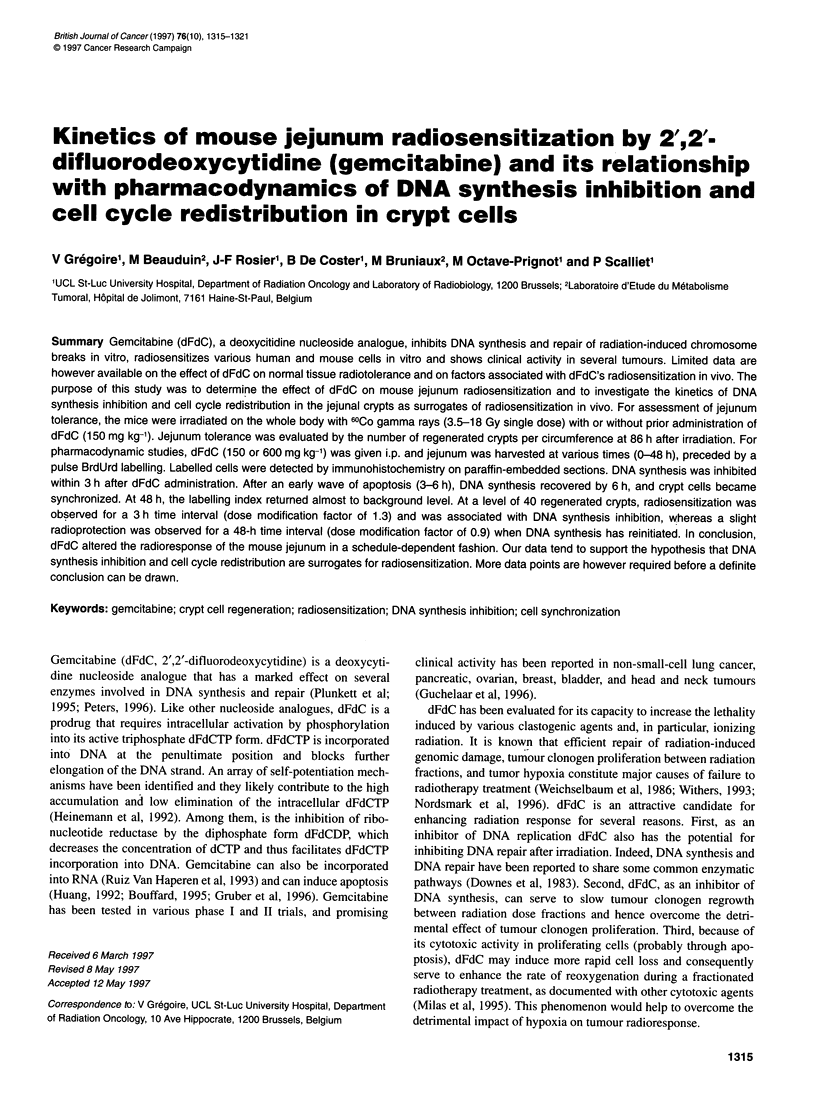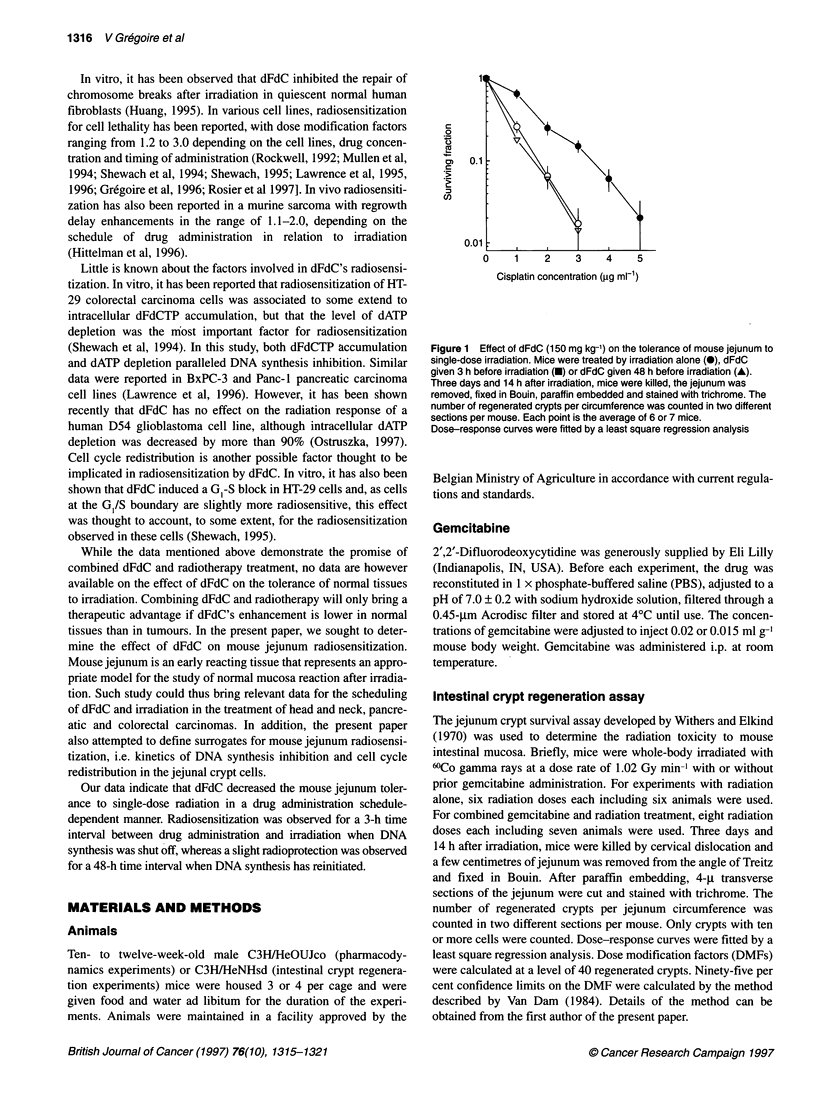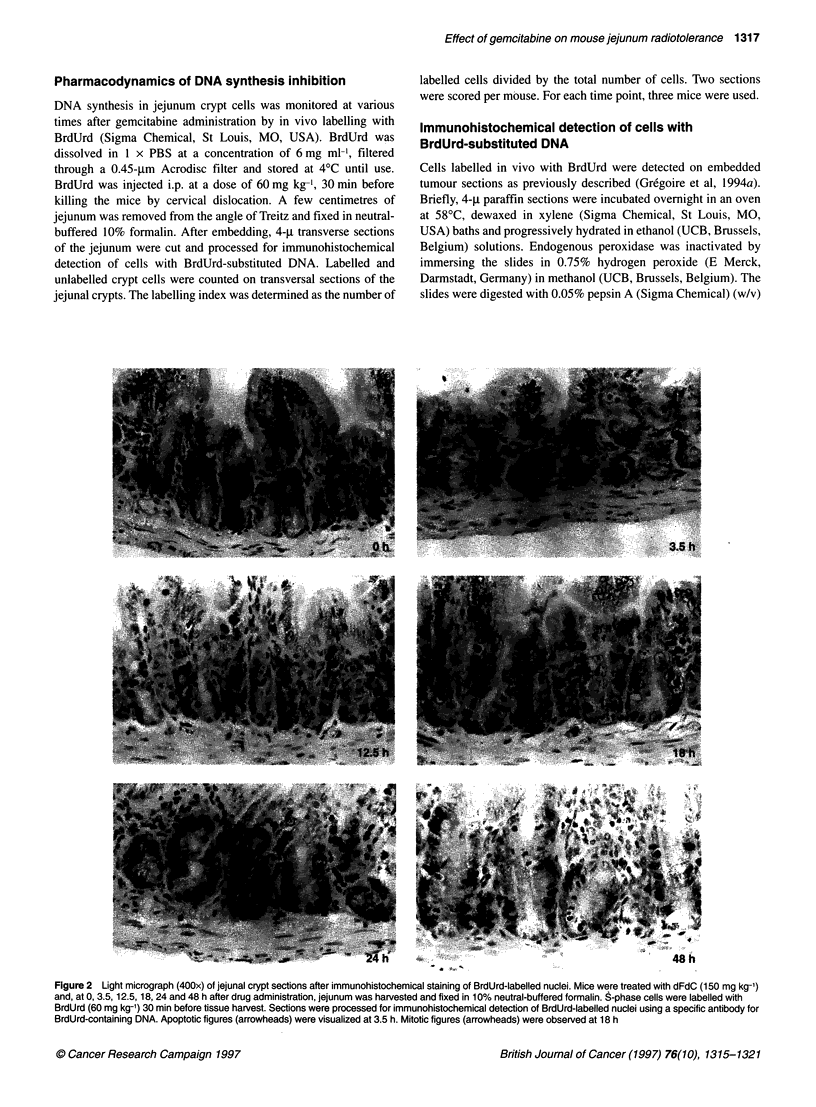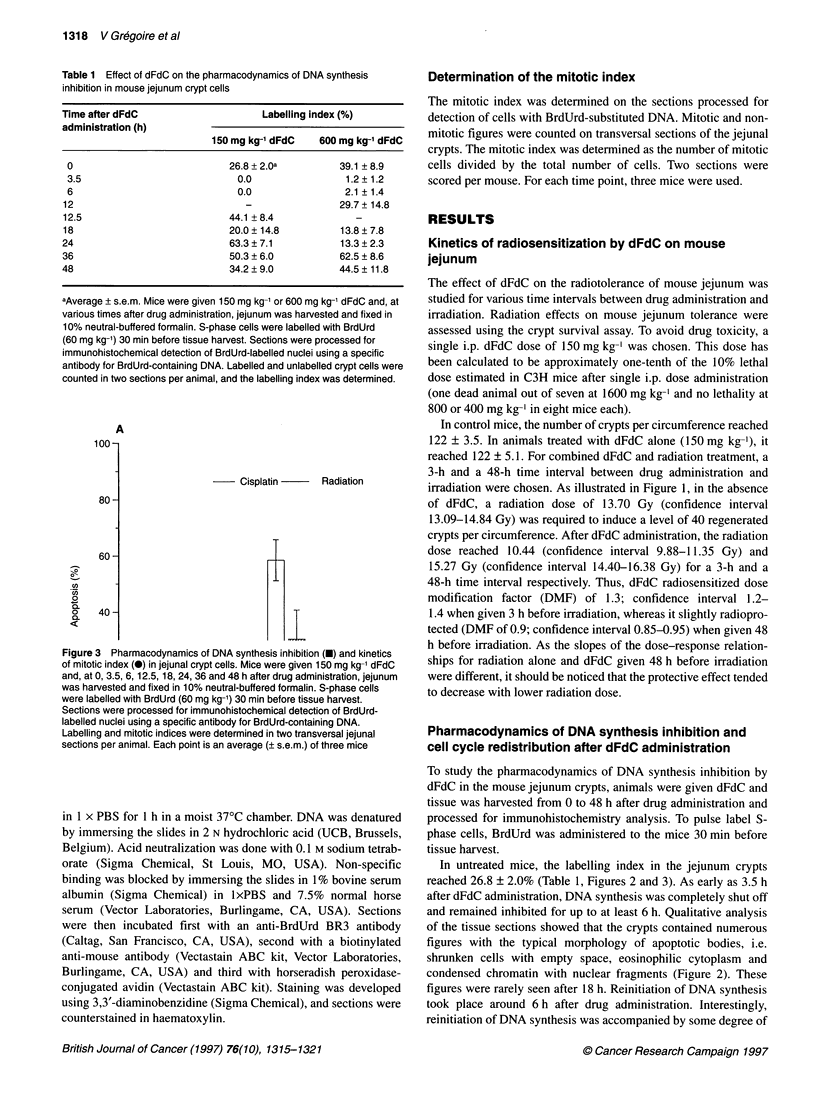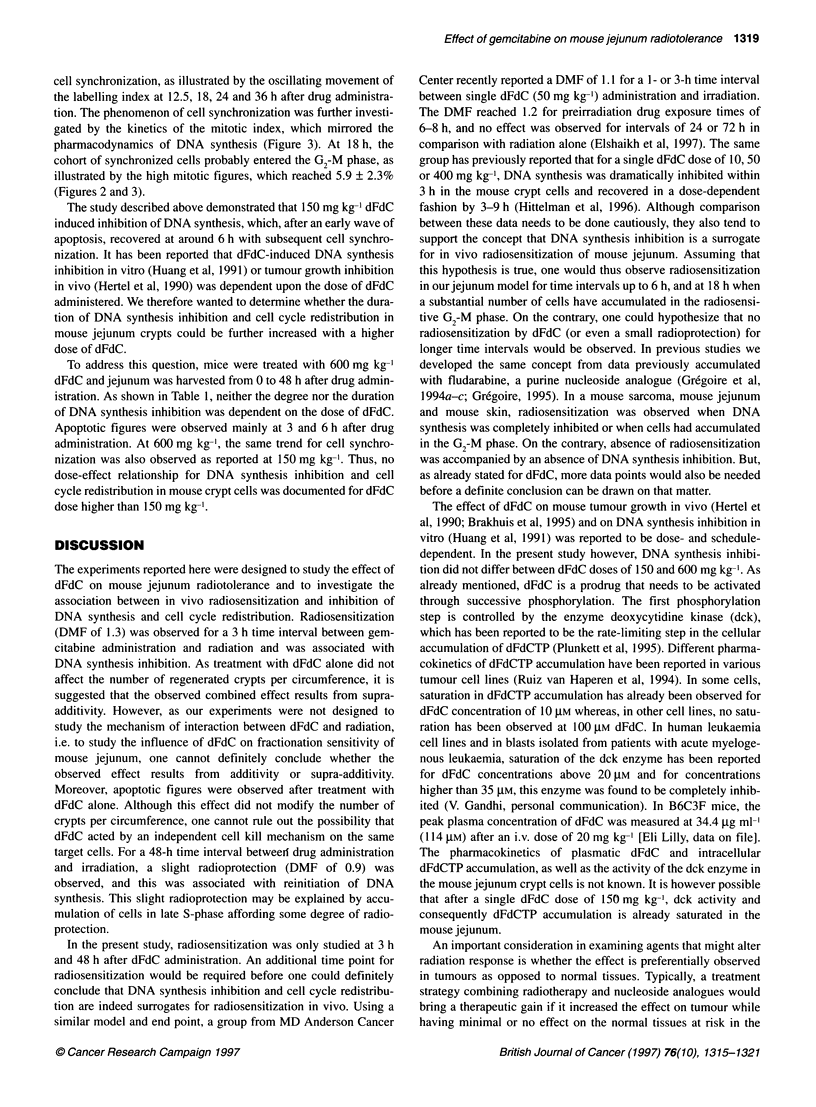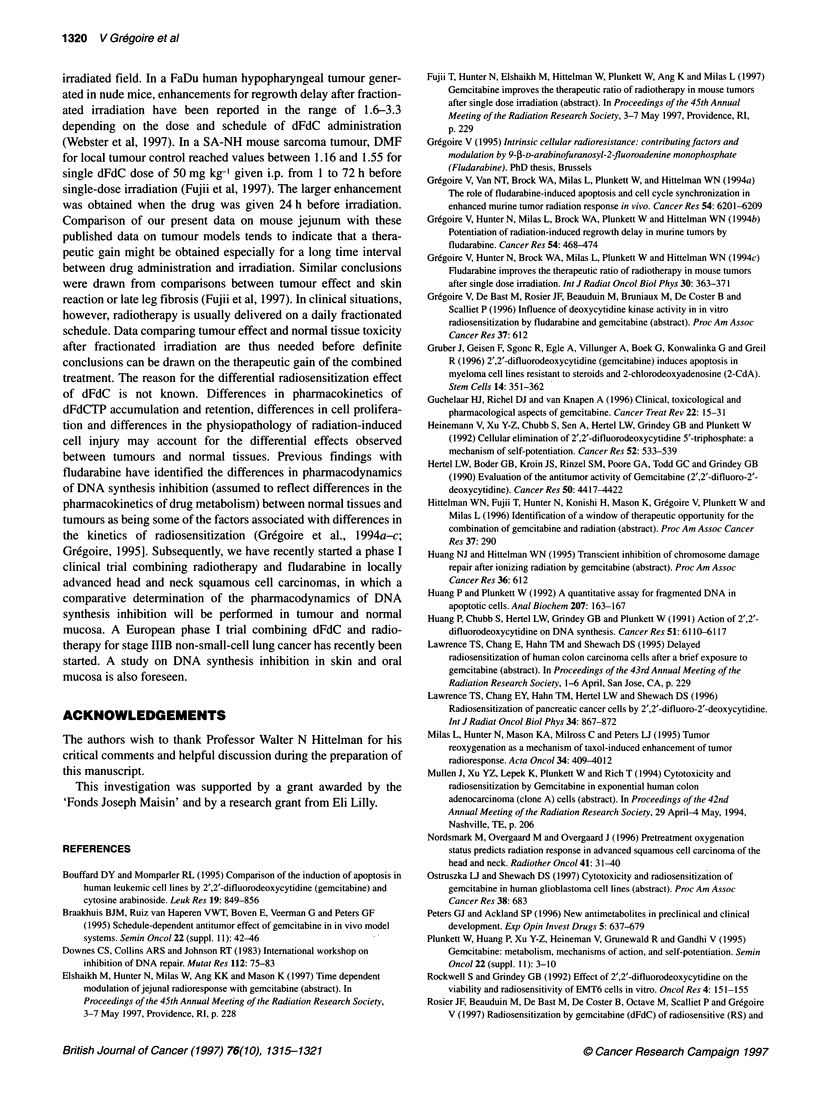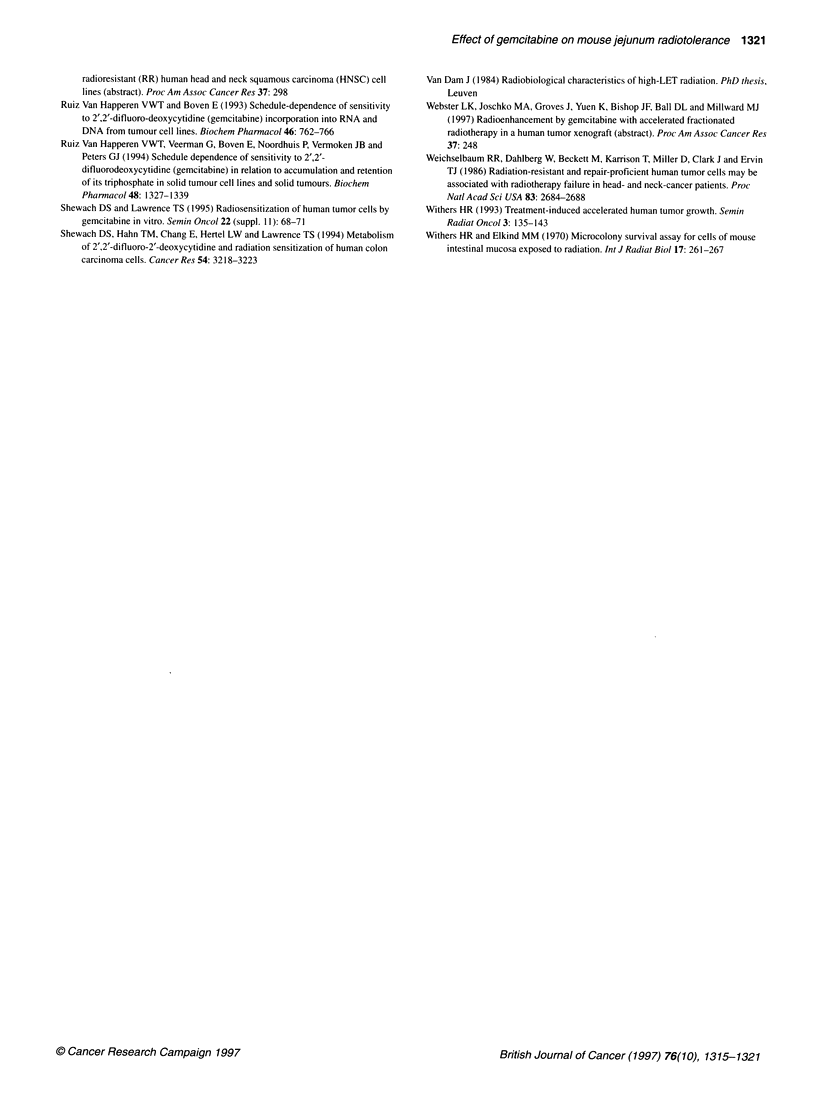# Kinetics of mouse jejunum radiosensitization by 2',2'-difluorodeoxycytidine (gemcitabine) and its relationship with pharmacodynamics of DNA synthesis inhibition and cell cycle redistribution in crypt cells.

**DOI:** 10.1038/bjc.1997.554

**Published:** 1997

**Authors:** V. GrÃ©goire, M. Beauduin, J. F. Rosier, B. De Coster, M. Bruniaux, M. Octave-Prignot, P. Scalliet

**Affiliations:** UCL St-Luc University Hospital, Department of Radiation Oncology, Brussels, Belgium.

## Abstract

**Images:**


British Journal of Cancer (1997) 76(10), 1315–1321
© 1997 Cancer Research Campaign

### Kinetics of mouse jejunum radiosensitization by 2′,2′-difluorodeoxycytidine (gemcitabine) and its relationship with pharmacodynamics of DNA synthesis inhibition and cell cycle redistribution in crypt cells

V Grégoire[1], M Beauduin[2], J-F Rosier[1], B De Coster[1], M Bruniaux[2], M Octave-Prignot[1] and P Scalliet[1]

[1]UCL St-Luc University Hospital, Department of Radiation Oncology and Laboratory of Radiobiology, 1200 Brussels; [2]Laboratoire d'Etude du Métabolisme Tumoral, Hôpital de Jolimont, 7161 Haine-St-Paul, Belgium

**Summary** Gemcitabine (dFdC), a deoxycitidine nucleoside analogue, inhibits DNA synthesis and repair of radiation-induced chromosome breaks in vitro, radiosensitizes various human and mouse cells in vitro and shows clinical activity in several tumours. Limited data are however available on the effect of dFdC on normal tissue radiotolerance and on factors associated with dFdC's radiosensitization in vivo. The purpose of this study was to determine the effect of dFdC on mouse jejunum radiosensitization and to investigate the kinetics of DNA synthesis inhibition and cell cycle redistribution in the jejunal crypts as surrogates of radiosensitization in vivo. For assessment of jejunum tolerance, the mice were irradiated on the whole body with $^{60}Co$ gamma rays (3.5–18 Gy single dose) with or without prior administration of dFdC (150 mg $kg^{-1}$). Jejunum tolerance was evaluated by the number of regenerated crypts per circumference at 86 h after irradiation. For pharmacodynamic studies, dFdC (150 or 600 mg $kg^{-1}$) was given i.p. and jejunum was harvested at various times (0–48 h), preceded by a pulse BrdUrd labelling. Labelled cells were detected by immunohistochemistry on paraffin-embedded sections. DNA synthesis was inhibited within 3 h after dFdC administration. After an early wave of apoptosis (3–6 h), DNA synthesis recovered by 6 h, and crypt cells became synchronized. At 48 h, the labelling index returned almost to background level. At a level of 40 regenerated crypts, radiosensitization was observed for a 3 h time interval (dose modification factor of 1.3) and was associated with DNA synthesis inhibition, whereas a slight radioprotection was observed for a 48-h time interval (dose modification factor of 0.9) when DNA synthesis has reinitiated. In conclusion, dFdC altered the radioresponse of the mouse jejunum in a schedule-dependent fashion. Our data tend to support the hypothesis that DNA synthesis inhibition and cell cycle redistribution are surrogates for radiosensitization. More data points are however required before a definite conclusion can be drawn.

**Keywords:** gemcitabine; crypt cell regeneration; radiosensitization; DNA synthesis inhibition; cell synchronization

Gemcitabine (dFdC, 2′,2′-difluorodeoxycytidine) is a deoxycytidine nucleoside analogue that has a marked effect on several enzymes involved in DNA synthesis and repair (Plunkett et al; 1995; Peters, 1996). Like other nucleoside analogues, dFdC is a prodrug that requires intracellular activation by phosphorylation into its active triphosphate dFdCTP form. dFdCTP is incorporated into DNA at the penultimate position and blocks further elongation of the DNA strand. An array of self-potentiation mechanisms have been identified and they likely contribute to the high accumulation and low elimination of the intracellular dFdCTP (Heinemann et al, 1992). Among them, is the inhibition of ribonucleotide reductase by the diphosphate form dFdCDP, which decreases the concentration of dCTP and thus facilitates dFdCTP incorporation into DNA. Gemcitabine can also be incorporated into RNA (Ruiz Van Haperen et al, 1993) and can induce apoptosis (Huang, 1992; Bouffard, 1995; Gruber et al, 1996). Gemcitabine has been tested in various phase I and II trials, and promising clinical activity has been reported in non-small-cell lung cancer, pancreatic, ovarian, breast, bladder, and head and neck tumours (Guchelaar et al, 1996).

Received 6 March 1997
Revised 8 May 1997
Accepted 12 May 1997

Correspondence to: V Grégoire, UCL St-Luc University Hospital, Department of Radiation Oncology, 10 Ave Hippocrate, 1200 Brussels, Belgium dFdC has been evaluated for its capacity to increase the lethality induced by various clastogenic agents and, in particular, ionizing radiation. It is known that efficient repair of radiation-induced genomic damage, tumour clonogen proliferation between radiation fractions, and tumor hypoxia constitute major causes of failure to radiotherapy treatment (Weichselbaum et al, 1986; Withers, 1993; Nordsmark et al, 1996). dFdC is an attractive candidate for enhancing radiation response for several reasons. First, as an inhibitor of DNA replication dFdC also has the potential for inhibiting DNA repair after irradiation. Indeed, DNA synthesis and DNA repair have been reported to share some common enzymatic pathways (Downes et al, 1983). Second, dFdC, as an inhibitor of DNA synthesis, can serve to slow tumour clonogen regrowth between radiation dose fractions and hence overcome the detrimental effect of tumour clonogen proliferation. Third, because of its cytotoxic activity in proliferating cells (probably through apoptosis), dFdC may induce more rapid cell loss and consequently serve to enhance the rate of reoxygenation during a fractionated radiotherapy treatment, as documented with other cytotoxic agents (Milas et al, 1995). This phenomenon would help to overcome the detrimental impact of hypoxia on tumour radioresponse.

In vitro, it has been observed that dFdC inhibited the repair of chromosome breaks after irradiation in quiescent normal human fibroblasts (Huang, 1995). In various cell lines, radiosensitization for cell lethality has been reported, with dose modification factors ranging from 1.2 to 3.0 depending on the cell lines, drug concentration and timing of administration (Rockwell, 1992; Mullen et al, 1994; Shewach et al, 1994; Shewach, 1995; Lawrence et al, 1995, 1996; Grégoire et al, 1996; Rosier et al 1997]. In vivo radiosensitization has also been reported in a murine sarcoma with regrowth delay enhancements in the range of 1.1–2.0, depending on the schedule of drug administration in relation to irradiation (Hittelman et al, 1996).

Little is known about the factors involved in dFdC's radiosensitization. In vitro, it has been reported that radiosensitization of HT-29 colorectal carcinoma cells was associated to some extend to intracellular dFdCTP accumulation, but that the level of dATP depletion was the most important factor for radiosensitization (Shewach et al, 1994). In this study, both dFdCTP accumulation and dATP depletion paralleled DNA synthesis inhibition. Similar data were reported in BxPC-3 and Panc-1 pancreatic carcinoma cell lines (Lawrence et al, 1996). However, it has been shown recently that dFdC has no effect on the radiation response of a human D54 glioblastoma cell line, although intracellular dATP depletion was decreased by more than 90% (Ostruszka, 1997). Cell cycle redistribution is another possible factor thought to be implicated in radiosensitization by dFdC. In vitro, it has also been shown that dFdC induced a $G_1$-S block in HT-29 cells and, as cells at the $G_1$/S boundary are slightly more radiosensitive, this effect was thought to account, to some extent, for the radiosensitization observed in these cells (Shewach, 1995).

While the data mentioned above demonstrate the promise of combined dFdC and radiotherapy treatment, no data are however available on the effect of dFdC on the tolerance of normal tissues to irradiation. Combining dFdC and radiotherapy will only bring a therapeutic advantage if dFdC's enhancement is lower in normal tissues than in tumours. In the present paper, we sought to determine the effect of dFdC on mouse jejunum radiosensitization. Mouse jejunum is an early reacting tissue that represents an appropriate model for the study of normal mucosa reaction after irradiation. Such study could thus bring relevant data for the scheduling of dFdC and irradiation in the treatment of head and neck, pancreatic and colorectal carcinomas. In addition, the present paper also attempted to define surrogates for mouse jejunum radiosensitization, i.e. kinetics of DNA synthesis inhibition and cell cycle redistribution in the jejunal crypt cells.

Our data indicate that dFdC decreased the mouse jejunum tolerance to single-dose radiation in a drug administration schedule-dependent manner. Radiosensitization was observed for a 3-h time interval between drug administration and irradiation when DNA synthesis was shut off, whereas a slight radioprotection was observed for a 48-h time interval when DNA synthesis has reinitiated.

### MATERIALS AND METHODS

### Animals

Ten- to twelve-week-old male C3H/HeOUJco (pharmacodynamics experiments) or C3H/HeNHsd (intestinal crypt regeneration experiments) mice were housed 3 or 4 per cage and were given food and water ad libitum for the duration of the experiments. Animals were maintained in a facility approved by the Belgian Ministry of Agriculture in accordance with current regulations and standards.

**Figure 1** Effect of dFdC (150 mg kg⁻¹) on the tolerance of mouse jejunum to single-dose irradiation. Mice were treated by irradiation alone (●), dFdC given 3 h before irradiation (■) or dFdC given 48 h before irradiation (▲). Three days and 14 h after irradiation, mice were killed, the jejunum was removed, fixed in Bouin, paraffin embedded and stained with trichrome. The number of regenerated crypts per circumference was counted in two different sections per mouse. Each point is the average of 6 or 7 mice. Dose–response curves were fitted by a least square regression analysis

### Gemcitabine

2′,2′-Difluorodeoxycytidine was generously supplied by Eli Lilly (Indianapolis, IN, USA). Before each experiment, the drug was reconstituted in 1 × phosphate-buffered saline (PBS), adjusted to a pH of 7.0 ± 0.2 with sodium hydroxide solution, filtered through a 0.45-μm Acrodisc filter and stored at 4°C until use. The concentrations of gemcitabine were adjusted to inject 0.02 or 0.015 ml $g^{-1}$ mouse body weight. Gemcitabine was administered i.p. at room temperature.

### Intestinal crypt regeneration assay

The jejunum crypt survival assay developed by Withers and Elkind (1970) was used to determine the radiation toxicity to mouse intestinal mucosa. Briefly, mice were whole-body irradiated with $^{60}Co$ gamma rays at a dose rate of 1.02 Gy $min^{-1}$ with or without prior gemcitabine administration. For experiments with radiation alone, six radiation doses each including six animals were used. For combined gemcitabine and radiation treatment, eight radiation doses each including seven animals were used. Three days and 14 h after irradiation, mice were killed by cervical dislocation and a few centimetres of jejunum was removed from the angle of Treitz and fixed in Bouin. After paraffin embedding, 4-μ transverse sections of the jejunum were cut and stained with trichrome. The number of regenerated crypts per jejunum circumference was counted in two different sections per mouse. Only crypts with ten or more cells were counted. Dose–response curves were fitted by a least square regression analysis. Dose modification factors (DMFs) were calculated at a level of 40 regenerated crypts. Ninety-five per cent confidence limits on the DMF were calculated by the method described by Van Dam (1984). Details of the method can be obtained from the first author of the present paper.

 © Cancer Research Campaign 1997

### Pharmacodynamics of DNA synthesis inhibition

DNA synthesis in jejunum crypt cells was monitored at various times after gemcitabine administration by in vivo labelling with BrdUrd (Sigma Chemical, St Louis, MO, USA). BrdUrd was dissolved in 1 × PBS at a concentration of 6 mg ml$^{-1}$, filtered through a 0.45-μm Acrodisc filter and stored at 4°C until use. BrdUrd was injected i.p. at a dose of 60 mg kg$^{-1}$, 30 min before killing the mice by cervical dislocation. A few centimetres of jejunum was removed from the angle of Treitz and fixed in neutral-buffered 10% formalin. After embedding, 4-μ transverse sections of the jejunum were cut and processed for immunohistochemical detection of cells with BrdUrd-substituted DNA. Labelled and unlabelled crypt cells were counted on transversal sections of the jejunal crypts. The labelling index was determined as the number of labelled cells divided by the total number of cells. Two sections were scored per mouse. For each time point, three mice were used.

### Immunohistochemical detection of cells with BrdUrd-substituted DNA

Cells labelled in vivo with BrdUrd were detected on embedded tumour sections as previously described (Grégoire et al, 1994*a*). Briefly, 4-μ paraffin sections were incubated overnight in an oven at 58°C, dewaxed in xylene (Sigma Chemical, St Louis, MO, USA) baths and progressively hydrated in ethanol (UCB, Brussels, Belgium) solutions. Endogenous peroxidase was inactivated by immersing the slides in 0.75% hydrogen peroxide (E Merck, Darmstadt, Germany) in methanol (UCB, Brussels, Belgium). The slides were digested with 0.05% pepsin A (Sigma Chemical) (w/v)

**Figure 2** Light micrograph (400×) of jejunal crypt sections after immunohistochemical staining of BrdUrd-labelled nuclei. Mice were treated with dFdC (150 mg kg$^{-1}$) and, at 0, 3.5, 12.5, 18, 24 and 48 h after drug administration, jejunum was harvested and fixed in 10% neutral-buffered formalin. S-phase cells were labelled with BrdUrd (60 mg kg$^{-1}$) 30 min before tissue harvest. Sections were processed for immunohistochemical detection of BrdUrd-labelled nuclei using a specific antibody for BrdUrd-containing DNA. Apoptotic figures (arrowheads) were visualized at 3.5 h. Mitotic figures (arrowheads) were observed at 18 h

© Cancer Research Campaign 1997

**Table 1** Effect of dFdC on the pharmacodynamics of DNA synthesis inhibition in mouse jejunum crypt cells

| Time after dFdC administration (h) | Labelling index (%) | |
|---|---|---|
| | 150 mg kg$^{-1}$ dFdC | 600 mg kg$^{-1}$ dFdC |
| 0 | 26.8 ± 2.0[a] | 39.1 ± 8.9 |
| 3.5 | 0.0 | 1.2 ± 1.2 |
| 6 | 0.0 | 2.1 ± 1.4 |
| 12 | – | 29.7 ± 14.8 |
| 12.5 | 44.1 ± 8.4 | – |
| 18 | 20.0 ± 14.8 | 13.8 ± 7.8 |
| 24 | 63.3 ± 7.1 | 13.3 ± 2.3 |
| 36 | 50.3 ± 6.0 | 62.5 ± 8.6 |
| 48 | 34.2 ± 9.0 | 44.5 ± 11.8 |

[a]Average ± s.e.m. Mice were given 150 mg kg$^{-1}$ or 600 mg kg$^{-1}$ dFdC and, at various times after drug administration, jejunum was harvested and fixed in 10% neutral-buffered formalin. S-phase cells were labelled with BrdUrd (60 mg kg$^{-1}$) 30 min before tissue harvest. Sections were processed for immunohistochemical detection of BrdUrd-labelled nuclei using a specific antibody for BrdUrd-containing DNA. Labelled and unlabelled crypt cells were counted in two sections per animal, and the labelling index was determined.

**Figure 3** Pharmacodynamics of DNA synthesis inhibition (■) and kinetics of mitotic index (●) in jejunal crypt cells. Mice were given 150 mg kg$^{-1}$ dFdC and, at 0, 3.5, 6, 12.5, 18, 24, 36 and 48 h after drug administration, jejunum was harvested and fixed in 10% neutral-buffered formalin. S-phase cells were labelled with BrdUrd (60 mg kg$^{-1}$) 30 min before tissue harvest. Sections were processed for immunohistochemical detection of BrdUrd-labelled nuclei using a specific antibody for BrdUrd-containing DNA. Labelling and mitotic indices were determined in two transversal jejunal sections per animal. Each point is an average (± s.e.m.) of three mice in 1 × PBS for 1 h in a moist 37°C chamber. DNA was denatured by immersing the slides in 2 N hydrochloric acid (UCB, Brussels, Belgium). Acid neutralization was done with 0.1 M sodium tetraborate (Sigma Chemical, St Louis, MO, USA). Non-specific binding was blocked by immersing the slides in 1% bovine serum albumin (Sigma Chemical) in 1×PBS and 7.5% normal horse serum (Vector Laboratories, Burlingame, CA, USA). Sections were then incubated first with an anti-BrdUrd BR3 antibody (Caltag, San Francisco, CA, USA), second with a biotinylated anti-mouse antibody (Vectastain ABC kit, Vector Laboratories, Burlingame, CA, USA) and third with horseradish peroxidase-conjugated avidin (Vectastain ABC kit). Staining was developed using 3,3′-diaminobenzidine (Sigma Chemical), and sections were counterstained in haematoxylin.

### Determination of the mitotic index

The mitotic index was determined on the sections processed for detection of cells with BrdUrd-substituted DNA. Mitotic and non-mitotic figures were counted on transversal sections of the jejunal crypts. The mitotic index was determined as the number of mitotic cells divided by the total number of cells. Two sections were scored per mouse. For each time point, three mice were used.

### RESULTS

### Kinetics of radiosensitization by dFdC on mouse jejunum

The effect of dFdC on the radiotolerance of mouse jejunum was studied for various time intervals between drug administration and irradiation. Radiation effects on mouse jejunum tolerance were assessed using the crypt survival assay. To avoid drug toxicity, a single i.p. dFdC dose of 150 mg kg$^{-1}$ was chosen. This dose has been calculated to be approximately one-tenth of the 10% lethal dose estimated in C3H mice after single i.p. dose administration (one dead animal out of seven at 1600 mg kg$^{-1}$ and no lethality at 800 or 400 mg kg$^{-1}$ in eight mice each).

In control mice, the number of crypts per circumference reached 122 ± 3.5. In animals treated with dFdC alone (150 mg kg$^{-1}$), it reached 122 ± 5.1. For combined dFdC and radiation treatment, a 3-h and a 48-h time interval between drug administration and irradiation were chosen. As illustrated in Figure 1, in the absence of dFdC, a radiation dose of 13.70 Gy (confidence interval 13.09–14.84 Gy) was required to induce a level of 40 regenerated crypts per circumference. After dFdC administration, the radiation dose reached 10.44 (confidence interval 9.88–11.35 Gy) and 15.27 Gy (confidence interval 14.40–16.38 Gy) for a 3-h and a 48-h time interval respectively. Thus, dFdC radiosensitized dose modification factor (DMF) of 1.3; confidence interval 1.2–1.4 when given 3 h before irradiation, whereas it slightly radioprotected (DMF of 0.9; confidence interval 0.85–0.95) when given 48 h before irradiation. As the slopes of the dose–response relationships for radiation alone and dFdC given 48 h before irradiation were different, it should be noticed that the protective effect tended to decrease with lower radiation dose.

### Pharmacodynamics of DNA synthesis inhibition and cell cycle redistribution after dFdC administration

To study the pharmacodynamics of DNA synthesis inhibition by dFdC in the mouse jejunum crypts, animals were given dFdC and tissue was harvested from 0 to 48 h after drug administration and processed for immunohistochemistry analysis. To pulse label S-phase cells, BrdUrd was administered to the mice 30 min before tissue harvest.

In untreated mice, the labelling index in the jejunum crypts reached 26.8 ± 2.0% (Table 1, Figures 2 and 3). As early as 3.5 h after dFdC administration, DNA synthesis was completely shut off and remained inhibited for up to at least 6 h. Qualitative analysis of the tissue sections showed that the crypts contained numerous figures with the typical morphology of apoptotic bodies, i.e. shrunken cells with empty space, eosinophilic cytoplasm and condensed chromatin with nuclear fragments (Figure 2). These figures were rarely seen after 18 h. Reinitiation of DNA synthesis took place around 6 h after drug administration. Interestingly, reinitiation of DNA synthesis was accompanied by some degree of

© Cancer Research Campaign 1997

cell synchronization, as illustrated by the oscillating movement of the labelling index at 12.5, 18, 24 and 36 h after drug administration. The phenomenon of cell synchronization was further investigated by the kinetics of the mitotic index, which mirrored the pharmacodynamics of DNA synthesis (Figure 3). At 18 h, the cohort of synchronized cells probably entered the $G_2$-M phase, as illustrated by the high mitotic figures, which reached 5.9 ± 2.3% (Figures 2 and 3).

The study described above demonstrated that 150 mg kg$^{-1}$ dFdC induced inhibition of DNA synthesis, which, after an early wave of apoptosis, recovered at around 6 h with subsequent cell synchronization. It has been reported that dFdC-induced DNA synthesis inhibition in vitro (Huang et al, 1991) or tumour growth inhibition in vivo (Hertel et al, 1990) was dependent upon the dose of dFdC administered. We therefore wanted to determine whether the duration of DNA synthesis inhibition and cell cycle redistribution in mouse jejunum crypts could be further increased with a higher dose of dFdC.

To address this question, mice were treated with 600 mg kg$^{-1}$ dFdC and jejunum was harvested from 0 to 48 h after drug administration. As shown in Table 1, neither the degree nor the duration of DNA synthesis inhibition was dependent on the dose of dFdC. Apoptotic figures were observed mainly at 3 and 6 h after drug administration. At 600 mg kg$^{-1}$, the same trend for cell synchronization was also observed as reported at 150 mg kg$^{-1}$. Thus, no dose-effect relationship for DNA synthesis inhibition and cell cycle redistribution in mouse crypt cells was documented for dFdC dose higher than 150 mg kg$^{-1}$.

### DISCUSSION

The experiments reported here were designed to study the effect of dFdC on mouse jejunum radiotolerance and to investigate the association between in vivo radiosensitization and inhibition of DNA synthesis and cell cycle redistribution. Radiosensitization (DMF of 1.3) was observed for a 3 h time interval between gemcitabine administration and radiation and was associated with DNA synthesis inhibition. As treatment with dFdC alone did not affect the number of regenerated crypts per circumference, it is suggested that the observed combined effect results from supra-additivity. However, as our experiments were not designed to study the mechanism of interaction between dFdC and radiation, i.e. to study the influence of dFdC on fractionation sensitivity of mouse jejunum, one cannot definitely conclude whether the observed effect results from additivity or supra-additivity. Moreover, apoptotic figures were observed after treatment with dFdC alone. Although this effect did not modify the number of crypts per circumference, one cannot rule out the possibility that dFdC acted by an independent cell kill mechanism on the same target cells. For a 48-h time interval between drug administration and irradiation, a slight radioprotection (DMF of 0.9) was observed, and this was associated with reinitiation of DNA synthesis. This slight radioprotection may be explained by accumulation of cells in late S-phase affording some degree of radioprotection.

In the present study, radiosensitization was only studied at 3 h and 48 h after dFdC administration. An additional time point for radiosensitization would be required before one could definitely conclude that DNA synthesis inhibition and cell cycle redistribution are indeed surrogates for radiosensitization in vivo. Using a similar model and end point, a group from MD Anderson Cancer Center recently reported a DMF of 1.1 for a 1- or 3-h time interval between single dFdC (50 mg kg$^{-1}$) administration and irradiation. The DMF reached 1.2 for preirradiation drug exposure times of 6–8 h, and no effect was observed for intervals of 24 or 72 h in comparison with radiation alone (Elshaikh et al, 1997). The same group has previously reported that for a single dFdC dose of 10, 50 or 400 mg kg$^{-1}$, DNA synthesis was dramatically inhibited within 3 h in the mouse crypt cells and recovered in a dose-dependent fashion by 3–9 h (Hittelman et al, 1996). Although comparison between these data needs to be done cautiously, they also tend to support the concept that DNA synthesis inhibition is a surrogate for in vivo radiosensitization of mouse jejunum. Assuming that this hypothesis is true, one would thus observe radiosensitization in our jejunum model for time intervals up to 6 h, and at 18 h when a substantial number of cells have accumulated in the radiosensitive $G_2$-M phase. On the contrary, one could hypothesize that no radiosensitization by dFdC (or even a small radioprotection) for longer time intervals would be observed. In previous studies we developed the same concept from data previously accumulated with fludarabine, a purine nucleoside analogue (Grégoire et al, 1994*a–c*; Grégoire, 1995). In a mouse sarcoma, mouse jejunum and mouse skin, radiosensitization was observed when DNA synthesis was completely inhibited or when cells had accumulated in the $G_2$-M phase. On the contrary, absence of radiosensitization was accompanied by an absence of DNA synthesis inhibition. But, as already stated for dFdC, more data points would also be needed before a definite conclusion can be drawn on that matter.

The effect of dFdC on mouse tumour growth in vivo (Hertel et al, 1990; Brakhuis et al, 1995) and on DNA synthesis inhibition in vitro (Huang et al, 1991) was reported to be dose- and schedule-dependent. In the present study however, DNA synthesis inhibition did not differ between dFdC doses of 150 and 600 mg kg$^{-1}$. As already mentioned, dFdC is a prodrug that needs to be activated through successive phosphorylation. The first phosphorylation step is controlled by the enzyme deoxycytidine kinase (dck), which has been reported to be the rate-limiting step in the cellular accumulation of dFdCTP (Plunkett et al, 1995). Different pharmacokinetics of dFdCTP accumulation have been reported in various tumour cell lines (Ruiz van Haperen et al, 1994). In some cells, saturation in dFdCTP accumulation has already been observed for dFdC concentration of 10 μM whereas, in other cell lines, no saturation has been observed at 100 μM dFdC. In human leukaemia cell lines and in blasts isolated from patients with acute myelogenous leukaemia, saturation of the dck enzyme has been reported for dFdC concentrations above 20 μM and for concentrations higher than 35 μM, this enzyme was found to be completely inhibited (V. Gandhi, personal communication). In B6C3F mice, the peak plasma concentration of dFdC was measured at 34.4 μg ml$^{-1}$ (114 μM) after an i.v. dose of 20 mg kg$^{-1}$ [Eli Lilly, data on file]. The pharmacokinetics of plasmatic dFdC and intracellular dFdCTP accumulation, as well as the activity of the dck enzyme in the mouse jejunum crypt cells is not known. It is however possible that after a single dFdC dose of 150 mg kg$^{-1}$, dck activity and consequently dFdCTP accumulation is already saturated in the mouse jejunum.

An important consideration in examining agents that might alter radiation response is whether the effect is preferentially observed in tumours as opposed to normal tissues. Typically, a treatment strategy combining radiotherapy and nucleoside analogues would bring a therapeutic gain if it increased the effect on tumour while having minimal or no effect on the normal tissues at risk in the

© Cancer Research Campaign 1997

irradiated field. In a FaDu human hypopharyngeal tumour generated in nude mice, enhancements for regrowth delay after fractionated irradiation have been reported in the range of 1.6–3.3 depending on the dose and schedule of dFdC administration (Webster et al, 1997). In a SA-NH mouse sarcoma tumour, DMF for local tumour control reached values between 1.16 and 1.55 for single dFdC dose of 50 mg $kg^{-1}$ given i.p. from 1 to 72 h before single-dose irradiation (Fujii et al, 1997). The larger enhancement was obtained when the drug was given 24 h before irradiation. Comparison of our present data on mouse jejunum with these published data on tumour models tends to indicate that a therapeutic gain might be obtained especially for a long time interval between drug administration and irradiation. Similar conclusions were drawn from comparisons between tumour effect and skin reaction or late leg fibrosis (Fujii et al, 1997). In clinical situations, however, radiotherapy is usually delivered on a daily fractionated schedule. Data comparing tumour effect and normal tissue toxicity after fractionated irradiation are thus needed before definite conclusions can be drawn on the therapeutic gain of the combined treatment. The reason for the differential radiosensitization effect of dFdC is not known. Differences in pharmacokinetics of dFdCTP accumulation and retention, differences in cell proliferation and differences in the physiopathology of radiation-induced cell injury may account for the differential effects observed between tumours and normal tissues. Previous findings with fludarabine have identified the differences in pharmacodynamics of DNA synthesis inhibition (assumed to reflect differences in the pharmacokinetics of drug metabolism) between normal tissues and tumours as being some of the factors associated with differences in the kinetics of radiosensitization (Grégoire et al., 1994*a–c*; Grégoire, 1995]. Subsequently, we have recently started a phase I clinical trial combining radiotherapy and fludarabine in locally advanced head and neck squamous cell carcinomas, in which a comparative determination of the pharmacodynamics of DNA synthesis inhibition will be performed in tumour and normal mucosa. A European phase I trial combining dFdC and radiotherapy for stage IIIB non-small-cell lung cancer has recently been started. A study on DNA synthesis inhibition in skin and oral mucosa is also foreseen.

### ACKNOWLEDGEMENTS

The authors wish to thank Professor Walter N Hittelman for his critical comments and helpful discussion during the preparation of this manuscript.

This investigation was supported by a grant awarded by the 'Fonds Joseph Maisin' and by a research grant from Eli Lilly.

### REFERENCES

Bouffard DY and Momparler RL (1995) Comparison of the induction of apoptosis in human leukemic cell lines by 2′,2′-difluorodeoxycytidine (gemcitabine) and cytosine arabinoside. *Leuk Res* **19**: 849–856

Braakhuis BJM, Ruiz van Haperen VWT, Boven E, Veerman G and Peters GF (1995) Schedule-dependent antitumor effect of gemcitabine in in vivo model systems. *Semin Oncol* **22** (suppl. 11): 42–46

Downes CS, Collins ARS and Johnson RT (1983) International workshop on inhibition of DNA repair. *Mutat Res* **112**: 75–83

Elshaikh M, Hunter N, Milas W, Ang KK and Mason K (1997) Time dependent modulation of jejunal radioresponse with gemcitabine (abstract). In *Proceedings of the 45th Annual Meeting of the Radiation Research Society*, 3–7 May 1997, Providence, RI, p. 228

Fujii T, Hunter N, Elshaikh M, Hittelman W, Plunkett W, Ang K and Milas L (1997) Gemcitabine improves the therapeutic ratio of radiotherapy in mouse tumors after single dose irradiation (abstract). In *Proceedings of the 45th Annual Meeting of the Radiation Research Society*, 3–7 May 1997, Providence, RI, p. 229

Grégoire V (1995) *Intrinsic cellular radioresistance: contributing factors and modulation by 9-β-D-arabinofuranosyl-2-fluoroadenine monophosphate (Fludarabine)*. PhD thesis, Brussels

Grégoire V, Van NT, Brock WA, Milas L, Plunkett W, and Hittelman WN (1994*a*) The role of fludarabine-induced apoptosis and cell cycle synchronization in enhanced murine tumor radiation response *in vivo*. *Cancer Res* **54**: 6201–6209

Grégoire V, Hunter N, Milas L, Brock WA, Plunkett W and Hittelman WN (1994*b*) Potentiation of radiation-induced regrowth delay in murine tumors by fludarabine. *Cancer Res* **54**: 468–474

Grégoire V, Hunter N, Brock WA, Milas L, Plunkett W and Hittelman WN (1994*c*) Fludarabine improves the therapeutic ratio of radiotherapy in mouse tumors after single dose irradiation. *Int J Radiat Oncol Biol Phys* **30**: 363–371

Grégoire V, De Bast M, Rosier JF, Beauduin M, Bruniaux M, De Coster B and Scalliet P (1996) Influence of deoxycytidine kinase activity in in vitro radiosensitization by fludarabine and gemcitabine (abstract). *Proc Am Assoc Cancer Res* **37**: 612

Gruber J, Geisen F, Sgonc R, Egle A, Villunger A, Boek G, Konwalinka G and Greil R (1996) 2′,2′-difluorodeoxycytidine (gemcitabine) induces apoptosis in myeloma cell lines resistant to steroids and 2-chlorodeoxyadenosine (2-CdA). *Stem Cells* **14**: 351–362

Guchelaar HJ, Richel DJ and van Knapen A (1996) Clinical, toxicological and pharmacological aspects of gemcitabine. *Cancer Treat Rev* **22**: 15–31

Heinemann V, Xu Y-Z, Chubb S, Sen A, Hertel LW, Grindey GB and Plunkett W (1992) Cellular elimination of 2′,2′-difluorodeoxycytidine 5′-triphosphate: a mechanism of self-potentiation. *Cancer Res* **52**: 533–539

Hertel LW, Boder GB, Kroin JS, Rinzel SM, Poore GA, Todd GC and Grindey GB (1990) Evaluation of the antitumor activity of Gemcitabine (2′,2′-difluoro-2′-deoxycytidine). *Cancer Res* **50**: 4417–4422

Hittelman WN, Fujii T, Hunter N, Konishi H, Mason K, Grégoire V, Plunkett W and Milas L (1996) Identification of a window of therapeutic opportunity for the combination of gemcitabine and radiation (abstract). *Proc Am Assoc Cancer Res* **37**: 290

Huang NJ and Hittelman WN (1995) Transcient inhibition of chromosome damage repair after ionizing radiation by gemcitabine (abstract). *Proc Am Assoc Cancer Res* **36**: 612

Huang P and Plunkett W (1992) A quantitative assay for fragmented DNA in apoptotic cells. *Anal Biochem* **207**: 163–167

Huang P, Chubb S, Hertel LW, Grindey GB and Plunkett W (1991) Action of 2′,2′-difluorodeoxycytidine on DNA synthesis. *Cancer Res* **51**: 6110–6117

Lawrence TS, Chang E, Hahn TM and Shewach DS (1995) Delayed radiosensitization of human colon carcinoma cells after a brief exposure to gemcitabine (abstract). In *Proceedings of the 43rd Annual Meeting of the Radiation Research Society*, 1–6 April, San Jose, CA, p. 229

Lawrence TS, Chang EY, Hahn TM, Hertel LW and Shewach DS (1996) Radiosensitization of pancreatic cancer cells by 2′,2′-difluoro-2′-deoxycytidine. *Int J Radiat Oncol Biol Phys* **34**: 867–872

Milas L, Hunter N, Mason KA, Milross C and Peters LJ (1995) Tumor reoxygenation as a mechanism of taxol-induced enhancement of tumor radioresponse. *Acta Oncol* **34**: 409–4012

Mullen J, Xu YZ, Lepek K, Plunkett W and Rich T (1994) Cytotoxicity and radiosensitization by Gemcitabine in exponential human colon adenocarcinoma (clone A) cells (abstract). In *Proceedings of the 42nd Annual Meeting of the Radiation Research Society*, 29 April–4 May, 1994, Nashville, TE, p. 206

Nordsmark M, Overgaard M and Overgaard J (1996) Pretreatment oxygenation status predicts radiation response in advanced squamous cell carcinoma of the head and neck. *Radiother Oncol* **41**: 31–40

Ostruszka LJ and Shewach DS (1997) Cytotoxicity and radiosensitization of gemcitabine in human glioblastoma cell lines (abstract). *Proc Am Assoc Cancer Res* **38**: 683

Peters GJ and Ackland SP (1996) New antimetabolites in preclinical and clinical development. *Exp Opin Invest Drugs* **5**: 637–679

Plunkett W, Huang P, Xu Y-Z, Heineman V, Grunewald R and Gandhi V (1995) Gemcitabine: metabolism, mechanisms of action, and self-potentiation. *Semin Oncol* **22** (suppl. 11): 3–10

Rockwell S and Grindey GB (1992) Effect of 2′,2′-difluorodeoxycytidine on the viability and radiosensitivity of EMT6 cells in vitro. *Oncol Res* **4**: 151–155

Rosier JF, Beauduin M, De Bast M, De Coster B, Octave M, Scalliet P and Grégoire V (1997) Radiosensitization by gemcitabine (dFdC) of radiosensitive (RS) and

 © Cancer Research Campaign 1997

radioresistant (RR) human head and neck squamous carcinoma (HNSC) cell lines (abstract). *Proc Am Assoc Cancer Res* **37**: 298

Ruiz Van Happeren VWT and Boven E (1993) Schedule-dependence of sensitivity to 2′,2′-difluoro-deoxycytidine (gemcitabine) incorporation into RNA and DNA from tumour cell lines. *Biochem Pharmacol* **46**: 762–766

Ruiz Van Happeren VWT, Veerman G, Boven E, Noordhuis P, Vermoken JB and Peters GJ (1994) Schedule dependence of sensitivity to 2′,2′-difluorodeoxycytidine (gemcitabine) in relation to accumulation and retention of its triphosphate in solid tumour cell lines and solid tumours. *Biochem Pharmacol* **48**: 1327–1339

Shewach DS and Lawrence TS (1995) Radiosensitization of human tumor cells by gemcitabine in vitro. *Semin Oncol* **22** (suppl. 11): 68–71

Shewach DS, Hahn TM, Chang E, Hertel LW and Lawrence TS (1994) Metabolism of 2′,2′-difluoro-2′-deoxycytidine and radiation sensitization of human colon carcinoma cells. *Cancer Res* **54**: 3218–3223

Van Dam J (1984) Radiobiological characteristics of high-LET radiation. *PhD thesis*, Leuven

Webster LK, Joschko MA, Groves J, Yuen K, Bishop JF, Ball DL and Millward MJ (1997) Radioenhancement by gemcitabine with accelerated fractionated radiotherapy in a human tumor xenograft (abstract). *Proc Am Assoc Cancer Res* **37**: 248

Weichselbaum RR, Dahlberg W, Beckett M, Karrison T, Miller D, Clark J and Ervin TJ (1986) Radiation-resistant and repair-proficient human tumor cells may be associated with radiotherapy failure in head- and neck-cancer patients. *Proc Natl Acad Sci USA* **83**: 2684–2688

Withers HR (1993) Treatment-induced accelerated human tumor growth. *Semin Radiat Oncol* **3**: 135–143

Withers HR and Elkind MM (1970) Microcolony survival assay for cells of mouse intestinal mucosa exposed to radiation. *Int J Radiat Biol* **17**: 261–267

© Cancer Research Campaign 1997