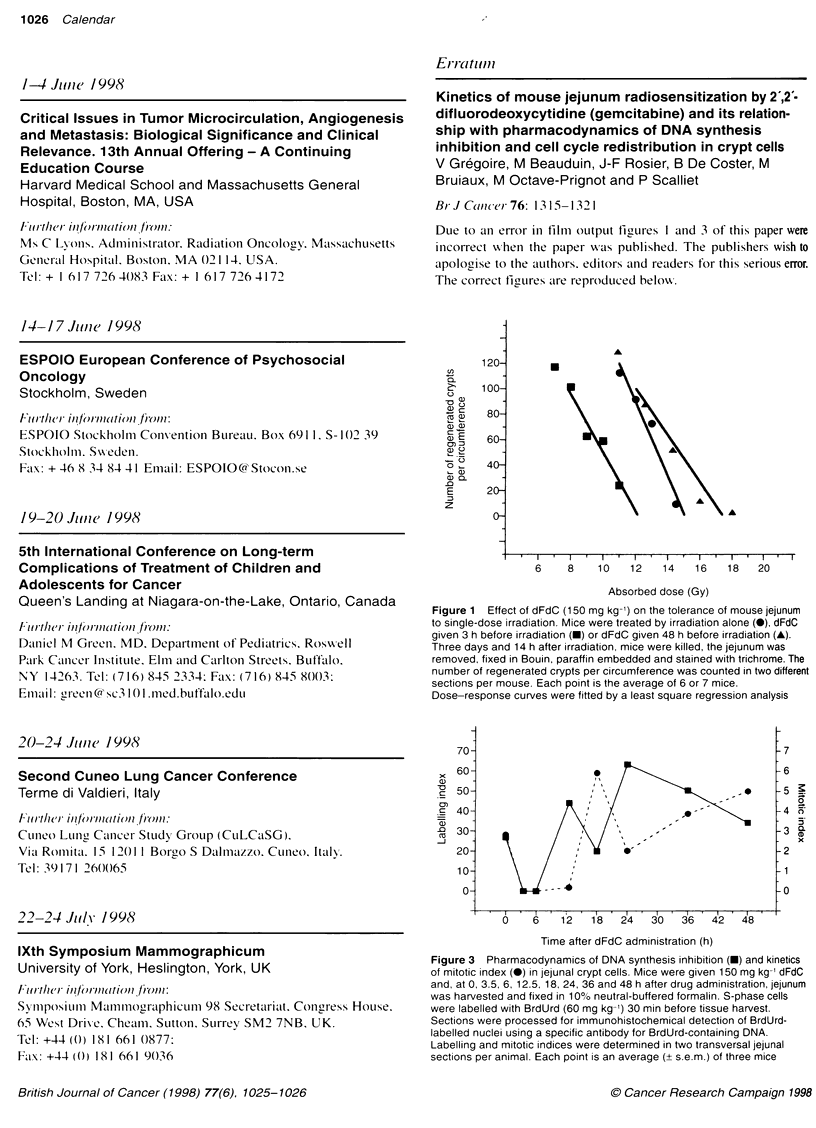# Kinetics of mouse jejunum radiosensitization by 2′,2′-difluorodeoxycytidine (gemcitabine) and its relationship with pharmacodynamics of DNA synthesis inhibition and cell cycle redistribution in crypt cells

**Published:** 1998-03

**Authors:** 


**Kinetics of mouse jejunum radiosensitization by 2′,2′-difluorodeoxycytidine (gemcitabine) and its relationship with pharmacodynamics of DNA synthesis inhibition and cell cycle redistribution in crypt cells**
V Grégoire, M Beauduin, J-F Rosier, B De Coster, M Bruiaux, M Octave-Prignot and P Scalliet

*Br J Cancer* **76**: 1315–1321

Due to an error in film output figures 1 and 3 of this paper were incorrect when the paper was published. The publishers wish to apologise to the authors, editors and readers for this serious error. The correct figures are reproduced below.

**Figure 1** Effect of dFdC (150 mg kg$^{-1}$) on the tolerance of mouse jejunum to single-dose irradiation. Mice were treated by irradiation alone (●), dFdC given 3 h before irradiation (■) or dFdC given 48 h before irradiation (▲). Three days and 14 h after irradiation, mice were killed, the jejunum was removed, fixed in Bouin, paraffin embedded and stained with trichrome. The number of regenerated crypts per circumference was counted in two different sections per mouse. Each point is the average of 6 or 7 mice. Dose–response curves were fitted by a least square regression analysis

**Figure 3** Pharmacodynamics of DNA synthesis inhibition (■) and kinetics of mitotic index (●) in jejunal crypt cells. Mice were given 150 mg kg$^{-1}$ dFdC and, at 0, 3.5, 6, 12.5, 18, 24, 36 and 48 h after drug administration, jejunum was harvested and fixed in 10% neutral-buffered formalin. S-phase cells were labelled with BrdUrd (60 mg kg$^{-1}$) 30 min before tissue harvest. Sections were processed for immunohistochemical detection of BrdUrd-labelled nuclei using a specific antibody for BrdUrd-containing DNA. Labelling and mitotic indices were determined in two transversal jejunal sections per animal. Each point is an average (± s.e.m.) of three mice

*© Cancer Research Campaign 1998*